# Reduction in the copy number and expression level of the recurrent human papillomavirus integration gene fragile histidine triad (*FHIT*) predicts the transition of cervical lesions

**DOI:** 10.1371/journal.pone.0175520

**Published:** 2017-04-17

**Authors:** Liming Wang, Hui Shen, Bei Feng, Da Zhu, Lan Yu, Xun Tian, Ci Ren, Chun Gao, Xiaomin Li, Ding Ma, Zheng Hu, Hui Wang

**Affiliations:** 1 Key Laboratory of Cancer Invasion and Metastasis of the Ministry of Education, Tongji Hospital, Tongji Medical College, Huazhong University of Science and Technology, Wuhan, Hubei, China; 2 Department of Obstetrics and Gynecology, Tongji Hospital, Tongji Medical College, Huazhong University of Science and Technology, Wuhan, Hubei, China; 3 Department of Obstetrics and Gynecology, The First Affiliated Hospital, Sun Yat-Sen University, Guangzhou, Guangdong, China; Georgetown University, UNITED STATES

## Abstract

Cervical cancer is the second most common cancer and the third leading cause of cancer death in females worldwide, especially in developing countries. High risk human papillomavirus (HR-HPV) infection causes cervical cancer and precancerous cervical intraepithelial neoplasia (CIN). Integration of the HR-HPV genome into the host chromatin is an important step in cervical carcinogenesis. The detection of integrated papillomavirus sequences-PCR (DIPS-PCR) allowed us to explore HPV integration in the human genome and to determine the pattern of this integration. We performed DIPS-PCR for 4 cell lines including 3 cervical cancer cell lines and 40 tissue samples. Overall, 32 HR-HPV integration loci were detected in the clinical samples and the HeLa and SiHa cell lines. Among all the integration loci, we identified three recurrent integration loci: 3p14.2 (3 samples), 13q22.1 (2 samples and a SiHa cell line) and 8q24 (1 sample and a HeLa cell line). To further explore the effect of HR-HPV integration in the 3p14.2 locus, we used fluorescence *in situ* hybridization (FISH) to determine the copy number of the 3p14.2 locus and immunohistochemistry (IHC) to determine the protein expression levels of the related *FHIT* gene in the clinical samples. Both the 3p14.2 locus copy number and FHIT protein expression levels showed significant decreases when CIN transitioned to cervical cancer. HPV copy number was also evaluated in these clinical samples, and the copy number of HPV increased significantly between CIN and cervical cancer samples. Finally, we employed receiver operating characteristic curve (ROC curve) analysis to evaluate the potential of all these indexes in distinguishing CIN and cervical cancer, and the HPV copy number, *FHIT* copy number and FHIT protein expression levels have good diagnostic efficiencies.

## Introduction

Persistent infection with High risk human papillomavirus (HR-HPV) is the main risk factor for the development of cervical intraepithelial neoplasia (CIN) and cervical carcinoma, but only 0.8% of HPV infections develop into cervical cancer. In China, HPV 16 and HPV 18 are the most prevalent types [1]. Infection with HPV is an early event in the multi-step cervical tumorigenesis. The oncogenic potential of HPV has been attributed mainly to the continued expression of E6 and E7 proteins [2]. The E6 and E7 oncogenes can cause inactivation of the tumor suppressor proteins p53 and pRB, respectively. This inactivation can drive normal cervical epithelial cells to immortalization and transformation[3]. *In vitro* models established by transfection of primary human keratinocytes with HPV 16 and HPV 18 have shown that cells can be immortalized by the action of the E6 and E7 genes [4]. There are still other genetic or environmental factors involved in cervical tumorigenesis [5]. Integration of the HR-HPV genome into a human chromosome ensures continued expression of the viral E6 and E7 oncogenes. Previous studies determined the primary genomic variation of cervical cancer and identified several hot spot integration loci [6, 7]. There is increasing evidence that HPV integration can occur in fragile sites of the human genome. A high frequency of allelic deletions on the short arm of 3p14.2 has been noted in cervical neoplasms [8]. This observation indicates that some tumor suppressor genes located in this region, including fragile histidine triad (*FHIT*), may play a role in cervical tumorigenesis. The *FHIT* gene is encoded by 10 exons in a 1.1-Kb transcript distributed over 1 Mb of genomic DNA, and aberrant *FHIT* transcripts were reported in cervical cancer. These aberrant transcripts were determined to correlate with cervical cancer outcomes [9].

In this study, we used DIPS-PCR and determined that the *FHIT* gene locus had the highest frequency of HPV integration in cervical cancer tissues. Then, with fluorescence *in situ* hybridization (FISH), we found that the *FHIT* copy number was lower in cervical cancer samples compared to CIN samples. Protein levels of FHIT were also reduced during CIN transition to cervical cancer as measured by an IHC assay.

## Materials and methods

### Tissue samples and data collection

37 CIN patients and 93 cervical cancer patients were recruited at Tongji Hospital between 2014 and 2016. As normal control, 9 patients in the gynecological department who were performed with hysterectomy and confirmed with normal cervix were also recruited. Samples were collected by surgery and biopsy at the same time. All pathology diagnoses were evaluated and confirmed by two different pathologists from the Pathology Department of Tongji Hospital. Among the patients with cervical lesions, 23 case of para-cancerous tissue were successfully collected. All patients signed the sample collection informed consent form. We had access to information that could identify individual participants after data collection. Tissue DNA extractions were performed for 40 cervical cancer samples and all CIN tissue samples, and 53 cervical cancer samples were formaldehyde-fixed and paraffin-embedded. Clinical data were obtained from individual patient charts. In this study, we used International Federation of Gynecology and Obstetrics (FIGO) staging. The tissue sample collection and experiments were supervised and approved by the Ethics Committee of Tongji Hospital.

### Tissue DNA extraction from clinical samples

Genomic DNA from clinical samples was extracted with a QIAGEN Blood & Tissue Kit (Cat No. 69504, Germany). The genomic DNA was dissolved in elution buffer, and its concentration was determined using a Nanodrop 2000 (Thermo Fisher ND 2000, USA); all the DNA samples were stored at -80°C.

### Detection of integrated papillomavirus sequences-PCR (DIPS-PCR)

The detection of integrated papillomavirus sequences by ligation mediated PCR (DIPS-PCR) was performed to determine the HPV integration loci at the DNA level. The assay was carried out following the method of *Luft et al* [3]. After digestion of the genomic DNA (0.6 μg) with 10 units of Sau3AI overnight, ligation of the Sau3AI-specific adapters (50 pmol) to the restricted genomic DNA was performed as described. The formation of the ds-adapter, comprising one short oligo (19 mer) and one long oligo (45 mer), is the most important step and was produced as described previously. The AP1 primer, which is specific for the adapter sequence, was used in the second PCR. The first round of PCR was linear and was performed in a total volume of 20 μL. The second round of PCR was exponential and was performed in a final volume of 50 μL with 2 μL of the linear PCR product as template. All the primers used in the procedure are listed in **S1 Table.** The second round exponential PCR products were analyzed on 2% agarose gels stained with Cyber Green and visualized using UV light in a ChemiImager 5500 (Alpha Innotech, USA). These PCR products were sequenced by Tianyi Biotechnology Ltd.

### Sequencing analysis of the PCR products

Sequencing results were analyzed using the UCSC database (http://genome.ucsc.edu) and NCBI nucleotide BLAST (http://blast.ncbi.nlm.nih.gov) with assembly hg19.

### Tissue Micro-Array (TMA) block construction

The Tissue Micro-Array blocks were constructed as follows. Hematoxylin and eosin–stained slides from all of the formalin-fixed, paraffin-embedded tissue blocks were determined to be representative of CIN and cervical cancer by a pathologist. From each block, one tissue biopsy (1-mm diameter) was placed on a recipient TMA block, 4 μm thick slides were obtained from the TMA block, and TMA slides were re-examined for the original lesion and used for the subsequent experiment.

### Fluorescence *in situ* hybridization (FISH)

A bacterial artificial chromosome (BAC) plasmid for *FHIT* (RP11-960D5) was purchased from Invitrogen. The whole genomic plasmid of the most common high-risk HPV type 16 (HPV 16) was a gift from Harald Zur Hausen. FISH probes were labeled by standard nick translation with digoxigenin-dUTPs. The TMA slides were dewaxed, dehydrated, and pre-treated with hydrogen peroxide, followed by rehydration, digestion with pepsin (1:3000) in HCl at 37°C, and dehydration. Probe and target DNA were denatured simultaneously prior to hybridization overnight. After hybridization, the preparations were washed stringently in formamide, 2×SSC. The probe was detected by peroxidase-conjugated sheep anti-digoxigenin Fab fragments (1:200; Roche) and then Cy3 or FITC labeled tyramide (1:50, Perkin Elmer) and was mounted in a Vectashield (Vector Laboratories) containing 4’,6-diamidino-2-phenylindole (DAPI). The results were analyzed using a fluorescence microscope (Olympus BX53) with cellSens fluorescence imaging system software. A minimum of 100 nuclei were scored for FISH copy number evaluation. The copy numbers were evaluated using punctate signal per nucleus. In order to gain precise calculation of copy numbers, the average signals per nucleus of *FHIT* were normalized to the average signal per nucleus found in para-cancerous biopsies and copy numbers in those samples without para-cancerous tissue were normalized to the average copy number of the 9 normal biopsies.

### Immunohistochemistry (IHC)

IHC reactions were performed on TMA slides following the standard protocol for 3,3’-Diaminobenzidine (DAB) staining. Briefly, slides were dewaxed, rehydrated, washed with water and then pre-treated in 3% hydrogen peroxide. Then, the slides were boiled with antigen retrieval solution and incubated with 1% BSA. The TMA slides were incubated overnight in a humidified chamber at 4°C with the primary antibody, a monoclonal anti-FHIT antibody (1:50, Abcam). A biotinylated antibody was applied, followed by streptavidin-peroxidase incubation. The expression staining was developed in a solution of 3,3’-Diaminobenzidine. Then, slides were counterstained with hematoxylin, dehydrated, cleared with xylene and mounted. Image pro plus (IPP) software was used to analyze the staining. IHC evaluation was based on the integral optical density (IOD) of staining intensity. The optical density means the absorbance of light in a unit area of the IHC figure, and the summation of the absorbance of an IHC figure makes integral optical density (IOD).

### Statistical analysis

Copy numbers and protein expression levels were compared with Student’s *t*-test and analysis of variance, and the Chi-square test was used to compare the expression level results. Receiver operating characteristic (ROC) curve analysis was used to evaluate the diagnostic capabilities of HPV copy number and *FHIT* copy number and protein expression levels. Maximum value of the Youden’s index may be used as a criterion for selecting the optimum cut-off point of numeric diagnostic biomarker.

## Results

### Breakpoints of the human genome and the HPV genome

**Fig 1:**
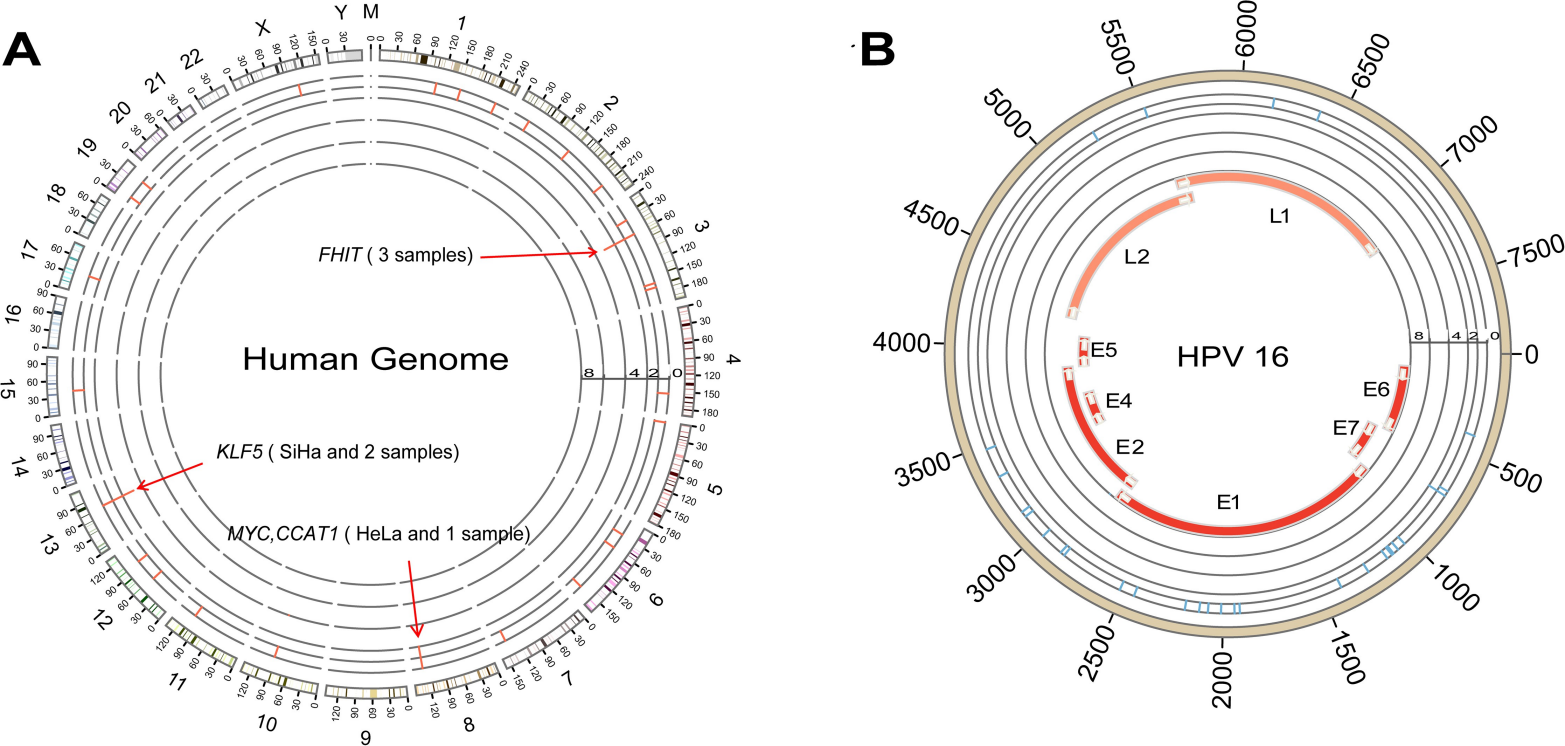
Integration loci of both the human genome and the HPV genome. (A) DIPS-PCR results show the breakpoints of the human genome, including chr8-128230632 in the HeLa cell line, chr13-74087562 in the SiHa cell line and 30 integration positions in 27 cervical cancer samples. (B) Breakpoints of the HPV genome. Most of the breaking loci are in *E1* and *E2*.

Genomic integration sites of HPV were detected by DIPS-PCR assay [3]. This protocol allowed the identification of human papillomavirus and human genome junctions. In the SiHa cell line, we detected the genomic chr13-74087562 breakpoint. This locus lies between the *KLF5* and *KLF12* genes. In the HeLa cell line, the breakpoint was identified as chr8-128230632, and this position lies within *CCAT1*, upstream of c-*MYC*. However, we did not detect any breakpoints in C33a and HEK293 cells. We further performed DIPS-PCR to identify the integration loci in 40 cervical cancer tissue samples and determined 30 integration positions in 27 cervical cancer samples, 13 of which were HPV negative. Among all the 30 integration loci of cervical cancer samples, most of the integration sites were located in introns or intergenic positions. Most integrated HR-HPV sequences were determined to be HPV 16 and only 1 were HPV 18 integration. The distribution of the integration breakpoints in the human genome and HPV genome are shown in **Fig 1**and **S2 Table.**

Notably, HPV integration into the 3p14.2 locus was detected in 3 samples, and these breakpoints are located in intron 4 of the *FHIT* gene.

As it was the most frequent integration locus, *FHIT* aroused our interest, further studies on *FHIT* are needed. To further evaluate the copy number and protein expression levels of the *FHIT* gene, we performed FISH and IHC. In addition, we assessed the potential of *FHIT* as a biomarker for the transition of CIN to cervical cancer.

### Copy number variation of HPV and *FHIT* and protein expression level variation of FHIT

We included 9 patients with normal cervical biopsy, 37 patients with CIN and 53 patients with cervical cancer in our study. The average age was 46.3, 47.1 and 43.3 years, respectively, the differences on age between these three groups were not significant. We evaluated cervical cancers using International Federation of Gynecology and Obstetrics (FIGO) staging.

**Fig 2:**
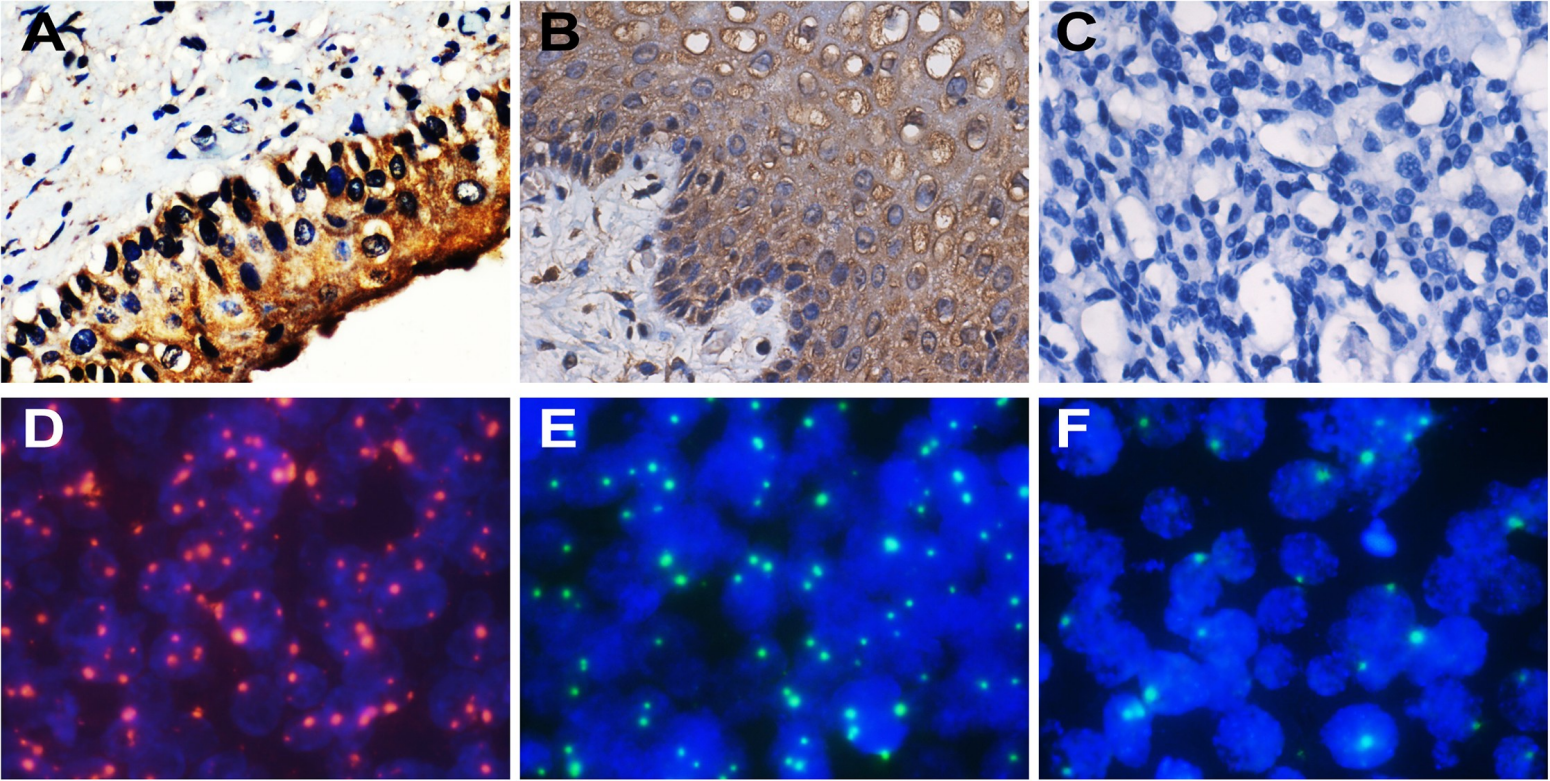
IHC staining of FHIT and FISH signals for HPV and *FHIT*. (A)-(C) typical FHIT staining in normal cervix (A), CIN (B) and cervical cancer (C) (×200). (D) HPV signals in cervical cancer (shown in red) (×1000). (E) and (F) *FHIT* signal variation in CIN (E) and cervical cancer (F) (shown in green) (×1000).

We analyzed the copy number variation of *FHIT* and HPV in normal cervix, CIN and cervical cancer by FISH. Typical FISH signal patterns are shown in **Fig 2.** We did not detect any HPV signals in normal cervical tissues.

**Fig 3:**
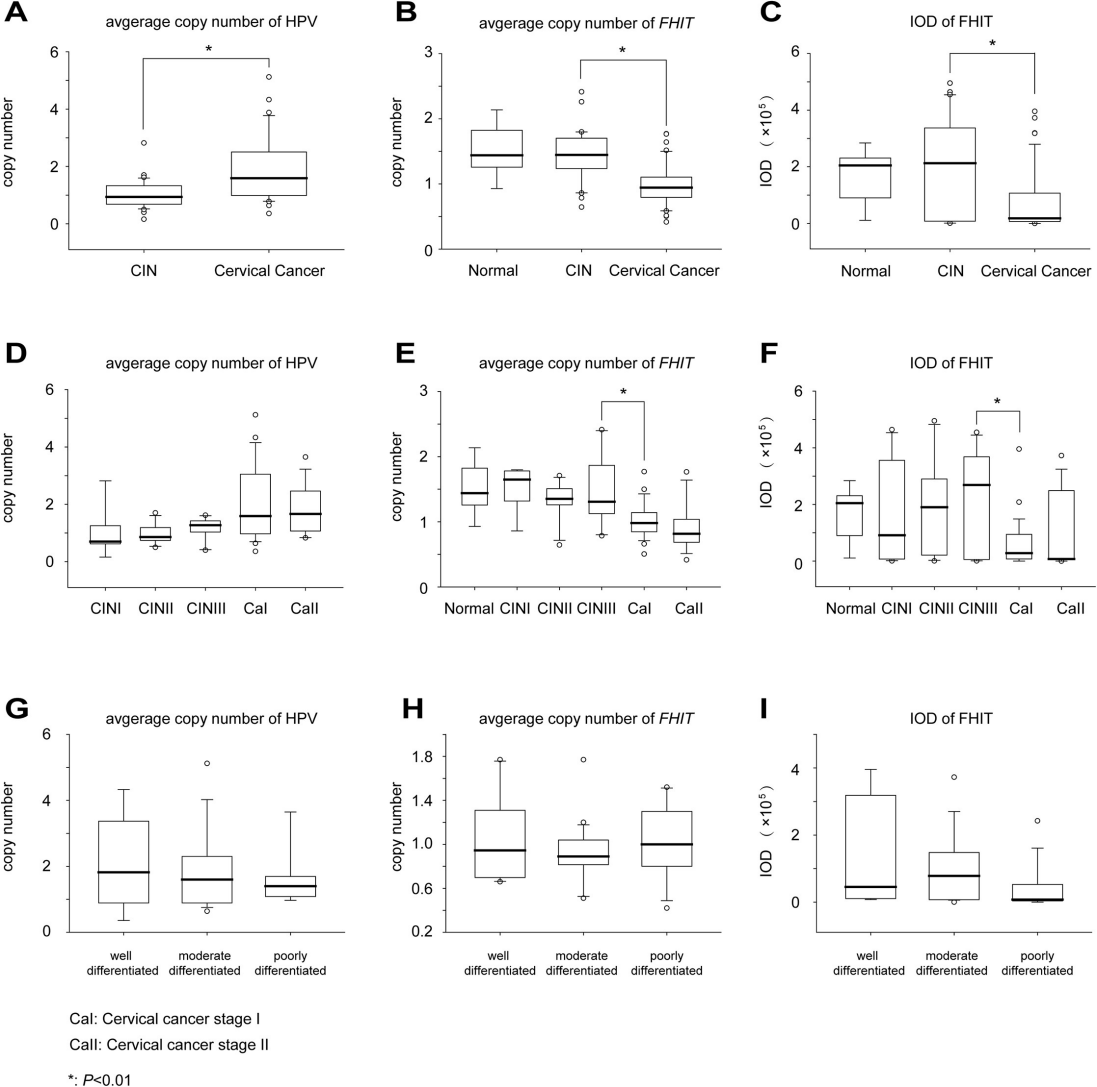
Variation of HPV copy number and *FHIT* copy number and protein expression levels. (A)-(C) All HPV copy numbers and *FHIT* copy numbers and protein expression levels show significant variation between CIN and cervical cancer. (D)-(F) HPV copy numbers and *FHIT* copy numbers and protein expression levels in different stages of cervical lesions. (G)-(I) HPV copy numbers and *FHIT* copy numbers and protein expression levels in different cervical cancer differentiation grades. The original data are shown in **S3 Table.**

For HPV copy number, in the normal cervical biopsies, we didn’t detect any HPV signals. An elevation was observed from CIN to cervical cancer (**Fig 3A**, 1.04 to 1.92, *P* < 0.001). However, there was no significant increase from CINI to CINII, CINII to CINIII, CINIII to cervical cancer stage I, or cervical cancer stage I to stage II (**Fig 3D**; *P* = 0.890; 0.197; 0.072 or 0.747). The differentiation of cervical cancer had no effect on the HPV copy number variation (**Fig 3G**, well differentiated to moderately differentiated, *P* = 0.693, and moderately differentiated to poorly differentiated, *P* = 0.624).

Generally, the average copy number of *FHIT* in cervical cancer was decreased compared to that of CIN (**Fig 3B**, 1.43 to 0.98, *P* < 0.001), but not from normal cervical samples to CIN (**Fig 3B**, 1.51 to 1.43, *P* = 0.595). However, between different stages of CINs and different stages of cervical cancers there was no particular aberrance (**Fig 3E**, CINI to CINII, *P* = 0.157; CINII to CINIII, *P* = 0.389; and cervical cancer stage I to stage II, *P* = 0.162). The most significant decline in average copy number was in the transition of CIN III to cancer stage I (**Fig 3E**, 1.49 to 1.01, *P* < 0.001). We determined that cervical cancer differentiation was not an influential factor for *FHIT* copy number (**Fig 3H**, well differentiated to moderately differentiated, *P* = 0.349, and moderately differentiated to poorly differentiated, *P* = 0.302)

IHC was used to analyze the protein expression levels of FHIT. Representative FHIT staining patterns are shown in **Fig 2**. Average IOD decreased significantly from CIN to cervical cancer (**Fig 3C**, 197112.59 to 79067.98, *P* < 0.001), but from normal cervical tissue to CIN, the change was not significant (**Fig 3C**, 166340.06 to 197112.59, *P* = 0.606). The most significant decrease was from CIN III to cancer stage I (**Fig 3F**, 223000.19 to 65783.97, *P* < 0.001), suggesting that FHIT is an important factor in the transition of CIN to cervical cancer. Transitions from CINI to CINII, from CINII to CINIII and from CaI to CaII do not have significant decreases (**Fig 3F**, P = 0.806; 0.655 and 0.287). Average IOD showed no significant differences between different grades of differentiation. (**Fig 3I**, well differentiated to moderately differentiated, *P* = 0.433, and moderately differentiated to poorly differentiated, *P* = 0.073).

To evaluate the potential of HPV copy number and *FHIT* copy number and protein expression level as biomarkers for diagnosis of CIN evolution to cervical cancer, receiver operating characteristic curve (ROC curve) analysis was employed. Cutoff points were made according to the maximum of Youden’s index. For HPV copy number, the area under the curve (AUC) was 0.769 (95% CI: 0.659–0.880), and the cutoff point was set to 1.54. Under such conditions, the sensitivity is 59.46%, and the specificity is 87.50%. The AUC of the *FHIT* copy number ROC was 0.820 (95% CI: 0.717–0.924). According to a cutoff point of 1.21, the sensitivity is 87.50%, and the specificity is 80.00%. For FHIT expression level, the AUC of the ROC curve was 0.683 (95% CI: 0.558–0.807), and the cutoff point was 161383.45. Under such conditions, the sensitivity is 85.11%, and the specificity is 55.89%.

## Discussion

Although there has been a large amount of research concentrating on HPV integration and cervical cancer, the mechanism of cervical cancer tumorigenesis still needs to be elucidated. Previous studies revealed that HPV tends to integrate near tumor associated genes, causing gene function alterations [10]. These alterations could be driving factors of cervical tumorigenesis. Also, an DNA segment deletion and insertion in the 8q24 locus close to the location where HPV 18 integrated into the human genome in the HeLa cell line have been reported [11]. These have implied genomic insertion and deletion may cause gene function dysregulation in cervical cancer carcinogenesis.

In our study, DIPS-PCR was used to detect the location of HPV integration in the human genome. In the SiHa and HeLa cell lines, we detected the genomic chr13-74087562 and chr8-128230632 breakpoints. This is consistent with the next generation sequencing results in our previous study [7]. In the cervical cancer samples, we have demonstrated that *FHIT* is one of the highest frequency loci for HPV integration. *FHIT*, or fragile histidine triad, is located at 3p14.2. FHIT is a target of tyrosine phosphorylation by the Src protein kinase, and the regulation of communication by Src may be important in the control of embryonic development and cellular growth [12]. FHIT also binds directly to the C-terminal domain of beta-catenin, a major component of the canonical Wnt signaling pathway, and represses transcription of target genes, including *cyclin D1*, *AXIN2*, *MMP14*, and survivin. Dysfunction of FHIT protein can cause overexpression of *cyclin D1* and other cell cycle genes. This can cause cell cycle disorder and carcinogenesis [13]. So *FHIT* works as a tumor suppressor genes. Studies showed reductions in the expression levels of FHIT have occurred in many kinds of malignancies, such as lung cancer [14], oropharyngeal squamous cell carcinomas [15], renal cell carcinomas [16] and colorectal carcinomas [17]. Terry G *et al* have reveal that loss of heterozygosity was an indicator of high-grade tumor, greater tumor depth and lymph node involvement [18]. Neyaz MK *et al* have identified a novel missense mutation of *FHIT* gene, this mutation can cause protein inactivation with loss of tumor suppressor activity [19]. Also, *FHIT* gene inactivation in cervical cancer was found to be strongly correlated with 5'-CpG island hypermethylation have been reported in previous study [20]. These findings suggested a crucial role of *FHIT* in cervical carcinogenesis.

Therefore, we investigated the potential of *FHIT* as a biomarker for cervical lesions. We assessed the copy number variation of the *FHIT* locus with FISH and the expression levels of the FHIT protein by IHC.

Previous report showed that methylation of *FHIT* was absent in CIN but was frequent in invasive tumor. Also, allelic losses, homozygous deletions, and aberrant *FHIT* transcripts are common in cervical cancers but occur at much lower frequencies in precancerous lesions [21]. This implied *FHIT* may play an important role in CIN evolution to cervical cancer. In our study, as CIN progressed to cervical cancer, *FHIT* copy number and protein expression levels both showed significant differences. The most significant reductions of *FHIT* copy numbers and FHIT expression were from CIN III to cancer stage I (**Fig 3B and 3F**), This finding indicated that there was obvious deletion in the *FHIT* gene locus when CIN progress into cervical cancer. And this deletion may cause protein expression decrease, suggesting that FHIT is an important factor in the transition of CIN to cervical cancer. We then explored the potential of them of being diagnostic biomarkers with ROC curve as well as HPV copy numbers. According to the ROC curve analysis, *FHIT* copy number has the most potential for being diagnostic biomarker compare to the HPV copy number and FHIT protein expression. The AUC of the ROC was 0.820 (95% CI: 0.717–0.924). According to a cutoff point of 1.21, the sensitivity is 87.50%, and the specificity is 80.00%.

All of the results suggested that *FHIT* may be a latent target of cervical cancer treatment, but in our study, integration of HPV into the *FHIT* gene was only detected in 3 samples, and *FHIT* copy number and expression levels decreased in a large proportion of the cervical cancer samples. HPV integration only explains part of this reduction. The reasons why the remainder of the samples also decreased in copy number and protein expression levels are still unknown. Further study is needed to determine the latent mechanism.

## Supporting information

S1 Table DIPS-PCR primers. DIPS-PCR primers for HPV 16 and HPV 18 and the formation of Sau3AI-specific adapters.
(PDF)

S2 Table DIPS-PCR results. **DIPS-PCR results of 40 cervical cancer samples.** 30 integration positions in 27 cervical cancer samples were found. Of all the breakpoints, *FHIT* was found to be a recurrent breakpoint.
(XLSX)

S3 Table Original data of Fig 3. (XLSX)

## References

[1] Yang H, Li LJ, Xie LX, Luo ZY, Lu M, Lin M, et al Clinical validation of a novel real-time human papillomavirus assay for simultaneous detection of 14 high-risk HPV type and genotyping HPV type 16 and 18 in China. Arch Virol. 2016;161(2):449–54. doi: 10.1007/s00705-015-2673-y 26577902 10.1007/s00705-015-2673-y

[2] von Knebel Doeberitz M, Rittmuller C, Aengeneyndt F, Jansen-Durr P, Spitkovsky D. Reversible repression of papillomavirus oncogene expression in cervical carcinoma cells: consequences for the phenotype and E6-p53 and E7-pRB interactions. J Virol. 1994;68(5):2811–21. PubMed Central PMCID: PMC236769. 8151752 10.1128/jvi.68.5.2811-2821.1994 PMC236769

[3] Schmitz M, Driesch C, Beer-Grondke K, Jansen L, Runnebaum IB, Durst M. Loss of gene function as a consequence of human papillomavirus DNA integration. Int J Cancer. 2012;131(5):E593–602. doi: 10.1002/ijc.27433 22262398 10.1002/ijc.27433

[4] Munger K, Phelps WC, Bubb V, Howley PM, Schlegel R. The E6 and E7 genes of the human papillomavirus type 16 together are necessary and sufficient for transformation of primary human keratinocytes. J Virol. 1989;63(10):4417–21. PubMed Central PMCID: PMC251060. 2476573 10.1128/jvi.63.10.4417-4421.1989 PMC251060

[5] Danaei G, Vander Hoorn S, Lopez AD, Murray CJ, Ezzati M, Comparative Risk Assessment collaborating g. Causes of cancer in the world: comparative risk assessment of nine behavioural and environmental risk factors. Lancet. 2005;366(9499):1784–93. doi: 10.1016/S0140-6736(05)67725-2 16298215 10.1016/S0140-6736(05)67725-2

[6] Ojesina AI, Lichtenstein L, Freeman SS, Pedamallu CS, Imaz-Rosshandler I, Pugh TJ, et al Landscape of genomic alterations in cervical carcinomas. Nature. 2014;506(7488):371–5. PubMed Central PMCID: PMC4161954. doi: 10.1038/nature12881 24390348 10.1038/nature12881 PMC4161954

[7] Hu Z, Zhu D, Wang W, Li W, Jia W, Zeng X, et al Genome-wide profiling of HPV integration in cervical cancer identifies clustered genomic hot spots and a potential microhomology-mediated integration mechanism. Nat Genet. 2015;47(2):158–63. doi: 10.1038/ng.3178 25581428 10.1038/ng.3178

[8] Wong YF, Chung TK, Cheung TH, Tam PO, Chang AM. Frequent loss of heterozygosity of chromosome 3 short arm detected by PCR-based microsatellite polymorphisms in cervical squamous cell carcinoma. Cancer Lett. 1997;115(2):161–4. 9149119 10.1016/s0304-3835(97)04723-x

[9] Yoshino K, Enomoto T, Nakamura T, Nakashima R, Wada H, Saitoh J, et al Aberrant FHIT transcripts in squamous cell carcinoma of the uterine cervix. Int J Cancer. 1998;76(2):176–81. 9537577 10.1002/(sici)1097-0215(19980413)76:2<176::aid-ijc2>3.0.co;2-u

[10] Roychowdhury A, Samadder S, Das P, Mandloi S, Addya S, Chakraborty C, et al Integrative genomic and network analysis identified novel genes associated with the development of advanced cervical squamous cell carcinoma. Biochim Biophys Acta. 2016;1861(1 Pt A):2899–911. 27641506 10.1016/j.bbagen.2016.09.014

[11] Adey A, Burton JN, Kitzman JO, Hiatt JB, Lewis AP, Martin BK, et al The haplotype-resolved genome and epigenome of the aneuploid HeLa cancer cell line. Nature. 2013;500(7461):207–11. PubMed Central PMCID: PMC3740412. doi: 10.1038/nature12064 23925245 10.1038/nature12064 PMC3740412

[12] Pichiorri F, Okumura H, Nakamura T, Garrison PN, Gasparini P, Suh SS, et al Correlation of fragile histidine triad (Fhit) protein structural features with effector interactions and biological functions. J Biol Chem. 2009;284(2):1040–9. PubMed Central PMCID: PMC2613607. doi: 10.1074/jbc.M806638200 19004824 10.1074/jbc.M806638200 PMC2613607

[13] Weiske J, Albring KF, Huber O. The tumor suppressor Fhit acts as a repressor of beta-catenin transcriptional activity. Proc Natl Acad Sci U S A. 2007;104(51):20344–9. PubMed Central PMCID: PMC2154433. doi: 10.1073/pnas.0703664105 18077326 10.1073/pnas.0703664105 PMC2154433

[14] Yu Y, Liu X, Yang Y, Zhao X, Xue J, Zhang W, et al Effect of FHIT loss and p53 mutation on HPV-infected lung carcinoma development. Oncol Lett. 2015;10(1):392–8. PubMed Central PMCID: PMC4487131. doi: 10.3892/ol.2015.3213 26171037 10.3892/ol.2015.3213 PMC4487131

[15] Gao G, Kasperbauer JL, Tombers NM, Wang V, Mayer K, Smith DI. A selected group of large common fragile site genes have decreased expression in oropharyngeal squamous cell carcinomas. Genes Chromosomes Cancer. 2014;53(5):392–401. doi: 10.1002/gcc.22150 24481768 10.1002/gcc.22150

[16] Ramp U, Caliskan E, Ebert T, Karagiannidis C, Willers R, Gabbert HE, et al FHIT expression in clear cell renal carcinomas: versatility of protein levels and correlation with survival. J Pathol. 2002;196(4):430–6. doi: 10.1002/path.1062 11920739 10.1002/path.1062

[17] Kapitanovic S, Cacev T, Loncar B, Catela Ivkovic T, Krizanac S, Pavelic K. Reduced FHIT expression is associated with tumor progression in sporadic colon adenocarcinoma. Exp Mol Pathol. 2014;96(1):92–7. doi: 10.1016/j.yexmp.2013.12.005 24370550 10.1016/j.yexmp.2013.12.005

[18] Terry G, Ho L, Londesborough P, Cross P, Lopes A, Monaghan J, et al The role of human papillomavirus type 16 and the fragile histidine triad gene in the outcome of cervical neoplastic lesions. British journal of cancer. 2004;91(12):2056–62. PubMed Central PMCID: PMC2409782. doi: 10.1038/sj.bjc.6602253 15570308 10.1038/sj.bjc.6602253 PMC2409782

[19] Neyaz MK, Hussain S, Hassan MI, Das BC, Husain SA, Bharadwaj M. Novel missense mutation in FHIT gene: interpreting the effect in HPV-mediated cervical cancer in Indian women. Molecular and cellular biochemistry. 2010;335(1–2):53–8. doi: 10.1007/s11010-009-0240-0 19730990 10.1007/s11010-009-0240-0

[20] Ki KD, Lee SK, Tong SY, Lee JM, Song DH, Chi SG. Role of 5'-CpG island hypermethylation of the FHIT gene in cervical carcinoma. Journal of gynecologic oncology. 2008;19(2):117–22. PubMed Central PMCID: PMC2676455. doi: 10.3802/jgo.2008.19.2.117 19471558 10.3802/jgo.2008.19.2.117 PMC2676455

[21] Virmani AK, Muller C, Rathi A, Zoechbauer-Mueller S, Mathis M, Gazdar AF. Aberrant methylation during cervical carcinogenesis. Clinical cancer research: an official journal of the American Association for Cancer Research. 2001;7(3):584–9. 11297252

