# Supplementary material for: Reduction in the copy number and expression level of the recurrent human papillomavirus integration gene fragile histidine triad (FHIT) predicts the transition of cervical lesions
Source: PLoS One. 2017 Apr 17;12(4):e0175520. doi: 10.1371/journal.pone.0175520 (PMC5393568; doi:10.1371/journal.pone.0175520)
Supplement: S1 Table — DIPS-PCR primers for HPV 16 and HPV 18 and the formation of Sau3AI-specific adapters. (PDF) [file pone.0175520.s001.pdf]

1.DIPS-PCR primers of HPV16

HPV16-1 first round	GGGATGTAATGGATGGTTTTATGT
HPV16-2 first round	GGCGCCATGAGACTGAAACACC
HPV16-3 first round	GCCAGAATGGATACAAAGACAAACA
HPV16-4 first round	TTTGCACGAGGACGAGGACA
HPV16-5 first round	AGAGCCAGACACCGGAAACC
HPV16-rev-1 first round	ACACAACAAACAACACTAATTCAACAT
HPV16-rev-2 first round	GCACCAAAGCCAGTATGAACC
HPV16-rev-3 first round	AGCACCTATAGATTTTCCACTACGAG
HPV16-rev-4 first round	AAGTTGGGTAGCCGATGCAC
HPV16-1 second round	GGGATGCTATATCAGATGACGAGAACG
HPV16-2 second round	GTGGAAGTGGGGGTGGTTGC
HPV16-3 second round	GGTACAATGGGCCTACGATAATGA
HPV16-4 second round	ACGAGGACAAGGAAAACGATGGAGA
HPV16-5 second round	GAAACCCCTGCCACACCACT
HPV16-rev-1 second round	TGAGGTGGTGGGTGTAGCTTT
HPV16-rev-2 second round	AATGGTGGACAATCACCTGGA
HPV16-rev-3 second round	AATGCCAGTACGCCTAGAGGTT
HPV16-rev-4 second round	CAGAACGTTTGTGTCGCATTG

1.DIPS-PCR primers of HPV18

HPV18-1 first round	CAGAAGGTACAGACGGGGAGG
HPV18-2 first round	TAGACAACGGGGGCACAGAGG
HPV18-3 first round	CACCAAAATTGCGAAGTAGTGT
HPV18-4 first round	AGGAAGAGGAAGATGCAGACACC
HPV18-5 first round	CCTACAGGCAACAACAAAAGACG
HPV18-rev-1 first round	TCCAGTATCTACCATATCACCATCTTCC
HPV18-rev-2 first round	TAGTGTCCACAGGCTCAAAGG
HPV18-rev-3 first round	TGGAAATAGACACAGAGGTAGACGAAG

HPV18-rev-4 first round	AGGGGACGTTATTACCACAATATACAC
HPV18-1 second round	GGGTTGTAACGGCTGGTTTTATGT
HPV18-2 second round	GGGCACAGAGGGCAACAACA
HPV18-3 second round	AATGGGAGACACACCTGAGTGGATACA
HPV18-4 second round	AGATGCAGACACCGAAGGAAACCC
HPV18-5 second round	AGGCAACAACAAAAGACGGAAACT
HPV18-rev-1 second round	ATCTACCATATCACCATCTTCCAAAACT
HPV18-rev-2 second round	GAGGATGGACGTGTAAGAAACTCAGG
HPV18-rev-3 second round	ATCAAACCCAGACGTGCCAGTAAA
HPV18-rev-4 second round	TACAGACAGATGGCAAAAGCGG

2.The primers used in the formation of Sau3AI-specific adapters

AL1	GGGCCATCAGTCAGCAGTCGTAGCCGGATCCAGACTT ACACGTTG
AP1	GGCCATCAGTCAGCAGTCGTAG
AS-Sau3AI	GATCCAACGTGTAAGTCTG-NH_2
